# Leprosy in Refugees and Migrants in Italy and a Literature Review of Cases Reported in Europe between 2009 and 2018

**DOI:** 10.3390/microorganisms8081113

**Published:** 2020-07-24

**Authors:** Anna Beltrame, Gianfranco Barabino, Yiran Wei, Andrea Clapasson, Pierantonio Orza, Francesca Perandin, Chiara Piubelli, Geraldo Badona Monteiro, Silvia Stefania Longoni, Paola Rodari, Silvia Duranti, Ronaldo Silva, Veronica Andrea Fittipaldo, Zeno Bisoffi

**Affiliations:** 1 Department of Infectious, Tropical Diseases and Microbiology, I.R.C.C.S. Sacro Cuore Don Calabria Hospital, Via Sempreboni 5, 37024 Negrar di Valpolicella, Italy; pierantonio.orza@sacrocuore.it (P.O.); francesca.perandin@sacrocuore.it (F.P.); chiara.piubelli@sacrocuore.it (C.P.); geraldo.monteiro@sacrocuore.it (G.B.M.); silvia.longoni@sacrocuore.it (S.S.L.); paola.rodari@sacrocuore.it (P.R.); silvia.duranti@sacrocuore.it (S.D.); ronaldo.silva@sacrocuore.it (R.S.); zeno.bisoffi@sacrocuore.it (Z.B.); 2 Dermatological Clinic, National Reference Center for Hansen’s Disease, Ospedale Policlinico San Martino, Sistema Sanitario Regione Liguria, Istituto di Ricovero e Cura a Carattere Scientifico per l’Oncologia, Largo Rosanna Benzi 10, 16132 Genoa, Italy; gianfranco.barabino@hsanmartino.it (G.B.); weiyiran0924@hotmail.com (Y.W.); clpndr@yahoo.com (A.C.); 3 Oncology Department, Mario Negri Institute for Pharmacological Research I.R.C.C.S., Via Giuseppe La Masa 19, 20156 Milano, Italy; vafittipaldo@hotmail.it; 4 Department of Diagnostic and Public Health, University of Verona, P.le L. A. Scuro 10, 37134 Verona, Italy

**Keywords:** Leprosy, *Mycobacterium leprae*, *Mycobacterium lepromatosis*, refugees, migrants, Italy, Europe

## Abstract

Leprosy is a chronic neglected infectious disease that affects over 200,000 people each year and causes disabilities in more than four million people in Asia, Africa, and Latin America. The disease can appear with a wide spectrum of clinical forms, and therefore the clinical suspicion is often difficult. Refugees and migrants from endemic countries affected by leprosy can remain undiagnosed in Europe due to the unpreparedness of clinicians. We retrospectively describe the characteristics of 55 refugees/migrants with a diagnosis of leprosy established in Italy from 2009 to 2018. Continents of origin were Africa (42%), Asia (40%), and South and Central America (18%). The symptoms reported were skin lesions (91%), neuropathy (71%), edema (7%), eye involvement (6%), fever (6%), arthritis (4%), and lymphadenopathy (4%). Seven patients (13%) had irreversible complications. Overall, 35% were relapses and 66% multibacillary leprosy. Furthermore, we conducted a review of 17 case reports or case series and five nationwide reports, published in the same decade, describing 280 migrant patients with leprosy in Europe. In Europe, leprosy is a rare chronic infectious disease, but it has not completely disappeared. Diagnosis and treatment of leprosy in refugees and migrants from endemic countries are a challenge. European guidelines for this neglected disease in this high-risk population would be beneficial.

## 1. Introduction

Leprosy, or Hansen’s disease, is a neglected tropical disease (NTD) with a long incubation period caused by *Mycobacterium leprae* (*M. leprae*) and *Mycobacterium lepromatosis (M. lepromatosis)* [1,2,3]. Leprosy occurs in a wide spectrum of clinical forms depending on the host cell-mediated immune (CMI) response to the pathogen from the tuberculoid pole, through to borderline cases ending at the lepromatous pole [4]. High CMI response is associated with a low number of bacilli (paucibacillary leprosy) and, inversely, low CMI response is connected with the presence of a high number of bacilli (multibacillary leprosy). If untreated, the chronic infection results in a progressive and permanent damage of the skin, peripheral nerves, and eyes, leading to physical deformities and disabilities [1,4].

Since 1982, the World Health Organization (WHO) has recommended multidrug therapy (MDT) based on a combination of dapsone–clofazimine–rifampicin, which has been distributed free of charge to all endemic countries since 1995 [5]. MDT and leprosy campaigns sponsored by the WHO worldwide have permitted a reduction in leprosy prevalence by over 90% [6]. However, 208,619 new leprosy cases were still reported globally at the end of 2018 [6]. About 95% of cases occurred in 23 high burden countries: Angola, Bangladesh, Brazil, Comoros, Democratic Republic of the Congo, Egypt, Ethiopia, Federated States of Micronesia, India, Indonesia, Ivory Coast, Kiribati, Madagascar, Mozambique, Myanmar, Nepal, Nigeria, Philippines, South Sudan, Sri Lanka, Sudan, Somalia, and the United Republic of Tanzania [6].

In Europe, leprosy incidence declined after the late medieval period with no cases reported to the WHO during the period 2009–2014, followed by an increased detection trend from 18 cases during 2015 to 50 cases at the end of 2018 [6], prevalently in foreign-born patients [7,8,9].

Italy, due to its geographic location, has long been an entrance door from the Middle East and Africa to Europe, up to the actual migratory crisis [10]. It is not surprising then that leprosy, a disease that in its most typical presentations was already known in the Roman Empire [11], is still being diagnosed in Italy, although in a fragmentary way [12,13,14]. A total of 1658 cases have been notified to the National Leprosy Register at the Ministry of Health during the period 1920–2018, with a decreasing trend from 847 cases (28.2 cases/year) during 1920–1949 to 307 (6.3 cases/year) during 1980–2018 [15]. In the 80s, the autochthonous leprosy foci of Liguria, Puglia, Sicily, and Sardinia became extinct [13,15]. Since then, cases have been reported in Italian expatriates living for long periods in endemic countries and in foreign residents, migrants, and refugees [15]. The proportion of cases observed in the latter population progressively increased from 18% (1970–1979) to 80% (2000–2006) [16], with a major component of irregular immigrants [14].

Leprosy has never been considered among the treatable infectious diseases to be screened in immigrants and refugees arriving in Europe, in contrast to tuberculosis, human immunodeficiency virus infection, hepatitis B, hepatitis C, parasitic infections (such as schistosomiasis and strongyloidiasis), and vaccine-preventable diseases (measles, mumps, rubella, diphtheria, tetanus, pertussis, poliomyelitis) [17,18].

## 2. Material and Methods

### 2.1. Study Design

This is a retrospective, observational study intended to described the epidemiology of leprosy diagnosed in refugees/migrants reported in two specialized centers in Northern Italy in a ten-year period (2009–2018) and review the literature on the cases published in Europe in the same period.

### 2.2. Ethics Statement

Ethical clearance was obtained from “Comitato Etico Provinciale di Verona e Rovigo”: protocol number 66506 of 25 November 2019). Informed consent was obtained from all subjects admitted to the two specialized centers included in the study. Parent or legal guardian consent was obtained for minors.

### 2.3. Study Population and Data Collection

The Dermatological Clinic, National Reference Center for Hansen’s Disease, Ospedale Policlinico San Martino, Sistema Sanitario Regione Liguria, Istituto di Ricovero e Cura a Carattere Scientifico per l’Oncologia, Genoa has observed more than 90% of leprosy cases in Italy since 1999. The Department of Infectious, Tropical Diseases, and Microbiology (DITM), Istituto di Ricovero e Cura a Carattere Scientifico Sacro Cuore Don Calabria Hospital of Negrar di Valpolicella (Verona) is a National Referral Center for Neglected Tropical and Parasitic Diseases and for cases of patients with rare infectious diseases in the Veneto Region. The analysis of the population followed by these two centers is therefore representative of the population of leprosy in migrants present in Italy.

We retrospectively collected data from the medical records of refugees/migrants with a diagnosis of leprosy admitted to the two centers between January 2009 and December 2018.

The following information were recorded in the study case report form (1) demographic characteristics of the patients (country of origin, gender and age); (2) duration of stay in Europe; (3) previous diagnosis and treatment of leprosy; (4) time elapsed from symptom onset to diagnosis; (5) clinical manifestations; (6) diagnostic methods used; (7) WHO classification [19]; (8) Ridley–Jopling classification [20]; (9) treatment (type and duration); and (10) outcome. Data were entered into a pre-designed Excel file and analyzed.

### 2.4. Review: Search Strategy and Selection Criteria

**Figure 1:**
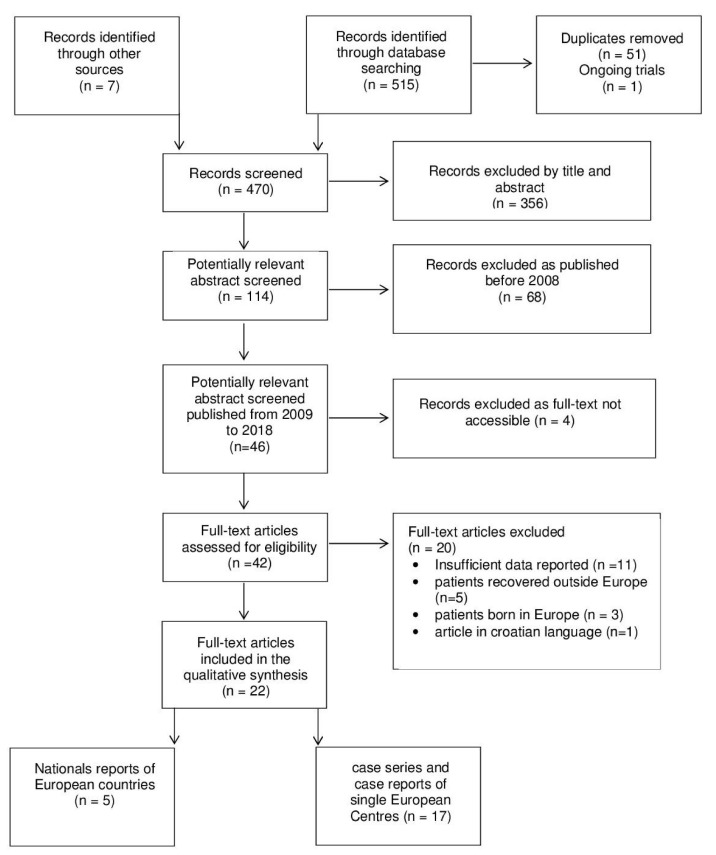
Study flow diagram. Information extracted from each paper were: (1) year of publication, (2) year and country of diagnosis, and (3) country of the patient’s origin. Other information was obtained when available. (4) Demographic characteristics of the patients (gender and age); (5) duration of stay in Europe; (6) clinical manifestations; (7) method of diagnosis; (8) WHO classification; (9) Ridley–Jopling classification.

We conducted a review of case reports and case series published between 2009 and 2018. No language restrictions were applied. Inclusion criteria were all case reports or case series reporting imported cases of leprosy diagnosed in Europe, upon the availability of main demographic and epidemiologic characteristics. Exclusion criteria were European patients. The following databases were searched for relevant studies: MEDLINE (PubMed), EMBASE (Embase.com), CENTRAL (Cochrane Library), Latin American and Caribbean Health Science Information Database (LILACS) (Bireme), and ClinicalTrials.gov (www.clinicaltrials.gov). The detailed electronic search strategy is reported in Appendix A. Figure 1 summarizes the search strategy as well as the references identified through other sources.

### 2.5. Definitions

The diagnosis of leprosy is made in the presence of one or more of the following criteria [4,19]:

- Clinical: definite loss of sensation in a pale (hypopigmented) or reddish skin patch (SP) or a thickened or enlarged peripheral nerve with loss of sensation and/or weakness of the muscles supplied by that nerve.
- Laboratory: demonstration of acid-fast bacilli (AFB) in a slit-skin smear (SSS) or in a skin biopsy (SB) of skin lesions with loss of sensation and/or nerve biopsy (NB) of thickened peripheral nerves.

Further diagnostic findings may include:

- Histopathology: evidence of granulomatous infiltrate.
- Biomolecular analysis: positive polymerase chain reaction (PCR) for *M. leprae* and/or *M. lepromatosis* in nasal swab, skin or nerve biopsy.

The WHO classification of leprosy includes [19]:

- Paucibacillary (PB) leprosy: a case of leprosy with one to five skin lesions, without a demonstrated presence of bacilli in a slit-skin smear
- Multibacillary (MB) leprosy: a case of leprosy with more than five skin lesions, or with nerve involvement (pure neuritis, or any number of skin lesions plus neuritis), or with the demonstrated presence of bacilli in a SSS, irrespective of the number of skin lesions.

The Ridley–Jopling classification of leprosy, based on clinical features and histopathology (CMI and bacterial load) include [20]:

- TT: Tuberculoid leprosy
- BT: Borderline tuberculoid leprosy
- BB: Mid-borderline leprosy
- BL: Borderline lepromatous leprosy
- LL: Lepromatous leprosy
- I: Indeterminate leprosy

The WHO-recommended treatment is either:

- Multidrug Therapy (MDT): rifampicin 600 mg monthly, dapsone 100 mg daily, clofazimine 300 mg monthly, and 50 mg daily (the treatment has to be continued until a negative SSS), or
- Rifampicin–Ofloxacina–Minocyclin (ROM) regimen: alternative treatment to clofazimine is based on Rifampicin 600 mg, Ofloxacin 400 mg (recently substituted by Moxifloxacin 400 mg), and Minocycline 100 mg, all once a month for 24 months.

### 2.6. Statistical Analysis

Descriptive statistics and plots were used to describe the characteristics of the entire cohort. Categorical variables were reported as absolute and relative frequencies, while quantitative variables were presented as mean and standard deviation or median and interquartile range (IQR), depending on the distribution of the variable. All estimations were reported with 95% confidence intervals. The Student’s t or Mann–Whitney U test was used to compare the means or medians between groups. Chi-square or Fisher exact test was used to compare the proportions. Statistical significance level was fixed at 5% (Bonferroni correction was used in case of multiple comparisons). Data analysis was performed with SAS software version 9.4 (Cary, NC, USA).

Our data analysis was further discussed using the results of the literature review and nationwide reports describing migrants with leprosy in European countries in the study period.

## 3. Results

### 3.1. Case Series

Sixty-five patients (57 migrants and eight Italians) with a diagnosis of leprosy were seen from January 2009 to December 2018. Demographic and epidemiologic data and clinical features were retrieved for 55 out of 57 migrants (Table 1). Two-thirds were males and the median age was 33 years. The continents of origin were Africa (42%), Asia (40%), and South and Central America (18%). The most reported countries were Sri Lanka (nine patients), Senegal (eight patients), Brazil (seven patients), Nigeria (six patients), and Philippines (five patients), followed by Bangladesh, Cuba, Egypt, India (three patients each country), Pakistan (two patients). Ivory Coast, Gambia, Ghana, Guinea, Morocco, and Togo contributed with one patient each.

The number of cases was relatively stable during the study period: nine cases in 2016; seven cases in 2009, in 2010, and in 2017; six cases in 2011, in 2014, and in 2018; three cases in 2015; and two cases in 2012 and in 2013. Most patients (48, or 87%) sought medical attention because of the symptoms while the others (with previous diagnosis) obtained the prescribed medications in order to continue the therapy in Italy. Nineteen patients (35%) reported a previous leprosy diagnosis and treatment and were classified as relapses.

**Figure 2:**
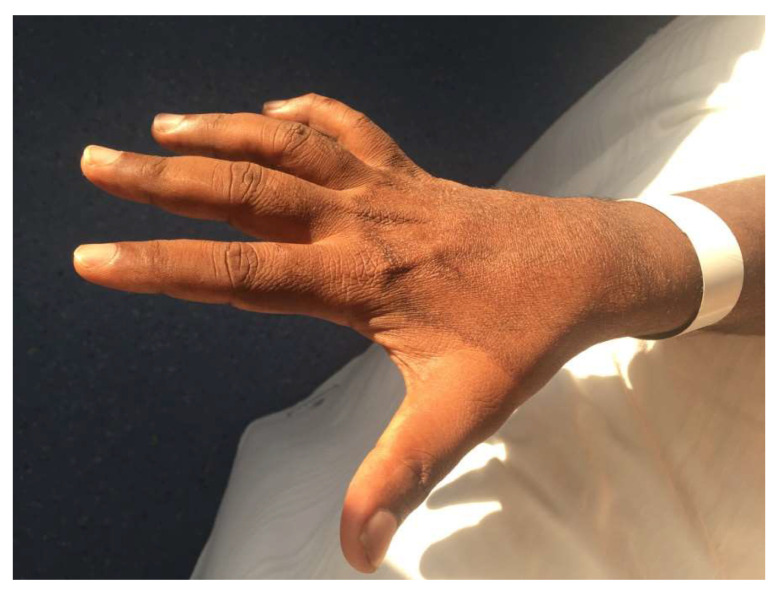
Skin lesion over the dorsum of hand in a 38-years old migrant from Sri Lanka with a diagnosis of borderline tuberculoid leprosy.

**Figure 3:**
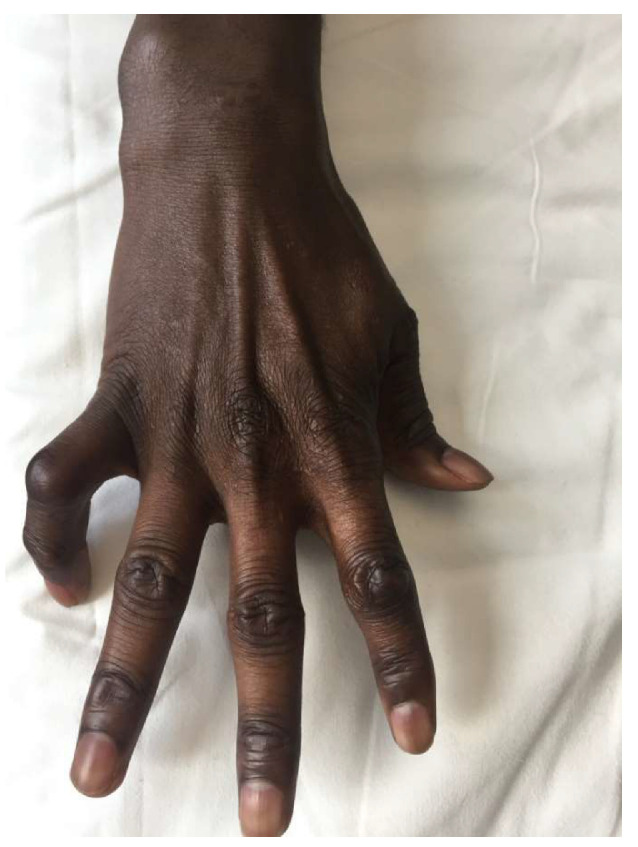
Hypotrophy of the hypothenar region with the flexion of the fifth proximal interphalangeal joint in a 37-years old migrant from Senegal with a diagnosis of tuberculoid leprosy.

The most frequently observed clinical findings were skin lesions (50 patients, 91%) (Figure 2) and neuropathy (39 patients, 71%) (Figure 3).

**Figure 4:**
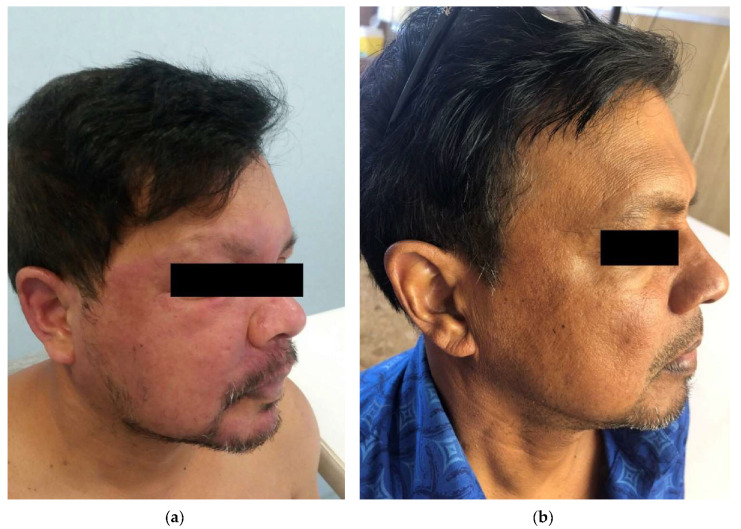
(**a**) Erythematous-infiltrated and edematous plaque edema symmetrically distributed over the face (with raised margins) and conjunctivitis (Type 1 reaction) in a 46-year old migrant from Bangladesh with a diagnosis of borderline lepromatous leprosy. (**b**) The plaque resolved after combination treatment with multidrug therapy and steroid.

An edema of the face (Figure 4a,b) or extremities was observed in four patients, an ocular involvement in three, while fever, arthritis, and lymphadenopathy were observed in two patients each.

**Figure 5:**
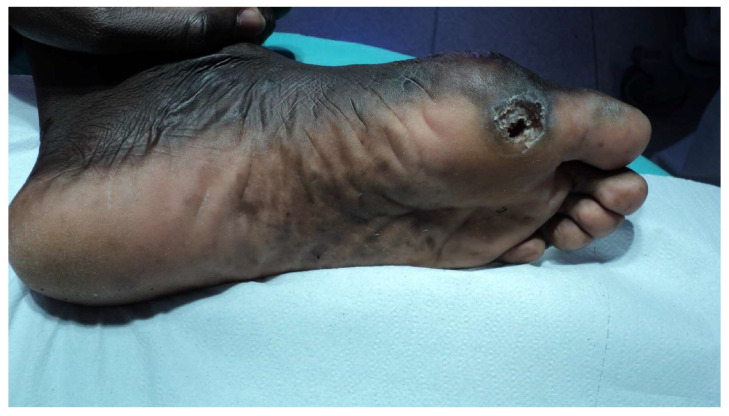
Plantar ulcer of the left foot caused by anesthesia of the sole resulting from damage to the posterior tibial nerve.

Seven patients had already developed some complications: claw deformity, ulcer (Figure 5), or amputation of fingers or motor deficit (reduced ability to use the hand) (Supplementary Video S1: deficit of abduction of the fifth finger), and difficulty in walking. The skin lesions were further differentiated (not reported in table) in skin patches (24/50 patients, 48%) (Figure 2), papules or nodules (21 patients, 42%), erythema nodosum (five patients, 10%), or ulcers (one patient, 2%). Most of the patches, papules, and nodules were multiple (100%, 79%, and 95%, respectively). The body areas predominantly of interest were (for the 42 patients for whom the data were available): the extremities (27/42), the face (13/42), and the trunk (3/42). In one case, the skin lesions affected the whole body.

Diagnosis. Twenty patients (37% of the 54 who submitted to a nasal swab) were acid-fast bacilli positive, and so were 39 of the 43 patients submitted to slit-skin smear (71%), and 13 of the 43 patients submitted to skin or nerve biopsy (30%). Histological findings of the 43 biopsies highlighted a granulomatous infiltrate in 21 patients (49%) and a lymphocitic or lympho-histiocytic infiltrate in 19 (44%), while three biopsies (7%) were lacking any patent pathological finding. The PCR for *M. leprae* and *M. lepromatosis* was performed on 24 patients (on SSS or biopsy), resulting in positive in 17 (85%). A single patient from Cuba with a diagnosis of diffuse lepromatous leprosy associated with Lucio’s phenomenon had a positive PCR for *M. lepromatosis* [21].

According to the WHO classification, 19 patients (34.5%) were diagnosed as paucibacillary leprosy and 36 (65.5%) as multibacillary leprosy. Based on the Ridley–Jopling classification, three patients (5%) were diagnosed as TT, 27 (49%) as BT, five (9%) as BL, 17 (31%) as LL, and three (5%) as BL/LL.

Fifty-two patients (95%) were treated with the MDT MB combination and three patients (5%) with ROM. The median duration of the therapy (for the patients who completed the treatment) was 12 months (IQR 12–24 months).

The outcome was as follows: 40 patients (73%) successfully completed the treatment, for seven (13%) this was still underway, while eight patients (14%) were lost to follow-up. Forty-seven patients (86%) lived with a variable number of household contacts, for the others, this information was not available.

### 3.2. The Review

Nineteen papers published between 2009 and 2018 were included in the review, accounting for 280 patients reported by five nationwide reports [7,8,9,22,23] (Table 2), 14 case reports [24,25,26,27,28,29,30,31,32,33,34,35,36,37], and three case series described by single centers [7,38,39] (Table 3).

Naturally, the chronology of the published cases is not the same as that of our case series, as many papers referred to cases observed years before the publication. The majority of the papers were from Italy [7,25,27,28,31,35,37], Spain [9,29,30,38,39], and the United Kingdom (UK) [26,32,33], whereas Portugal [22], France [24], Germany [34], Greece [23], and Denmark [8] contributed with one paper each. The continents of origin were South and Central America for 144 patients (51%), Africa (79 patients, 28%), and Asia (55 patients, 20%). For two patients, theses details were missing.

For 37 migrants for whom the demographic details were available (Table 3), males accounted for two thirds of the cases (24 or 65%). The median age was 28 years (range 11–65 years); the minors were five (age from 11 to 15 years). A large proportion of migrants presented with skin lesions (33 patients, 89%) and neuropathy (16 patients, 43%), prevalently of the ulnar nerve. Other manifestations were fever (five patients, 13.5%), arthritis or synovitis (four patients, 11%), lymphadenopathy (four patients, 11%), infiltration of the face (four patients, 11%), edema of face or extremities (three patients, 8%), hair loss or madarosis (two patients, 5%), diffuse rash (two patients, 5%), eye involvement (one patient, 3%). Three patients (8%) already had an irreversible claw deformity.

## 4. Discussion

We report the main epidemiological and clinical characteristics of 55 cases of leprosy treated in Italy in the decade from 2009 to 2018. In parallel, we reviewed all European cases and case series published in the same decade. The latter of course encompasses a broader timeframe, as many of the cases published were observed earlier than 2009.

Most of the published European cases were diagnosed in Latin American (52%) and African (28%) patients. In contrast, our study population mainly originated from Africa (42%) and Asia (40%). The origin of the published European cases was not uniform across countries, however, while 56% of the cases observed in Portugal were from Africa [22], and 87% and 50% of the cases observed in Denmark and Greece, respectively, were from Asia [8,23]; while in Spain, Latin American patients predominated (72%) [9].

The relatively small number of leprosy reported in Europe and in Italy is in contrast with the increasing wave of migration of the last few decades from endemic countries [10,40,41]. In 1994, the Italian Ministry of Health recognized four national hospitals as reference centers for the diagnosis and cure of leprosy (Genoa, Gioia del Colle, Messina e Cagliari), and in 1999, the Italian guidelines for the control of Hansen’s disease were published, indicating the Hospital of Genoa as the reference Italian laboratory for the diagnosis of leprosy (National Reference Center for Hansen Disease, NRCHD) [4]. However, leprosy cases are observed in all Italian regions and only subsequently are they are referred to the reference centers, mostly to NRCHD [14,15]. Therefore, all clinicians dealing with migrants should be able to recognize the symptoms and signs that may raise the suspicion of leprosy, particularly in high-risk populations.

Leprosy is extremely difficult to recognize and it is probably under-diagnosed for a number of reasons, the first one being the lack of knowledge, and therefore of clinical suspicion, by European clinicians. Only a few of them are trained to identify, for instance, the typical skin anesthetic lesions or peripheral nerve (i.e., ulnar) pathology [4]. Moreover, migrants may present with acute diseases such as malaria or with chronic infections that are much easier to suspect and diagnose such as tuberculosis, thus overshadowing the often much more subtle signs and symptoms of leprosy. Furthermore, leprosy can appear with other less common signs that can mimic other diseases [42]. This was also true for some of our patients who presented, for example, with edema of the face or the extremities, eye involvement, fever, arthritis, and lymphadenopathy, hardly attributable to leprosy. The diagnostic work-up is by no means simple, requiring trained experts, usually only available in referral centers, in order to implement the correct diagnostic procedures [4]. The diagnosis is based on a thorough clinical examination plus the combination of all the available tests, considering that their sensitivity is variable. In our patients, AFB was found only in 37% of nasal swab, 71% of SSS, and 30% of biopsies, while a granulomatous inflammation was reported in less than 50% of the cases. Molecular methods are indeed more sensitive, and in Europe, they should always be used on any swab and/or biopsy. In our patients, in the cases when it was performed (about half of the cases), the PCR resulted in being positive in 85%. A Cuban patient with diffuse generalized skin ulcerations caused by *M. lepromatosis* [21] highlights the need to differentiate the two species of Mycobacterium as they are probably not uncommon [3]. In fact, early diagnosis and full MDT treatment remain the cornerstone of leprosy control, even in non-endemic countries [6]. New guidelines for the diagnosis, treatment, and prevention of leprosy in endemic countries have been issued in 2018 by the WHO [19] and are specifically targeted to low and middle income countries. Unfortunately, no specific guidelines are yet available for the diagnosis and management of imported leprosy in non-endemic countries.

Leprosy is likely to be transmitted via droplets from the nose and the mouth, during close and frequent contacts with MB untreated patients. The first symptoms may appear after a long incubation period (5–10 years) with intermittent symptoms/signs that gradually worsen. In our study, migrant patients were MB in 66% of the cases, similar to the WHO data (60%). The proportion of MB cases indicates, indirectly, the amount of infection in the community. Over 85% of our patients lived in the same house with at least one person that should be considered at risk of acquisition of the infection. The operational manual for the Global Leprosy Strategy 2016–2020 also describes the procedures for contact screening in low endemic settings [43].

Notably, the Asiatic patients in our series were more often MB (77%), had a higher proportion of AFB positive nasal swab and SSS (52% and 82%, respectively) as well as granulomatous infiltrate in the biopsies (69%), and of positive DNA for *M. leprae* (89%). They also reported, on average, a briefer period of stay in Italy before the diagnosis, which may be assumed to indicate an easier access to the health system. The average latency between the appearance of signs and symptoms and the diagnosis was about 12 months in our series, shorter than that reported in the United Kingdom (1.8 years) [44]. However, in our African population, this reached 26 months. The reasons of this difference should be studied in order to improve the access to early diagnosis in this growing, generally young, high-risk population as barriers of language, culture, and/or law may have made the access to health care more problematic.

The WHO is actively promoting the health of refugees and migrants [45]. Leprosy should be diagnosed at the initial stage when the skin lesions are the predominant manifestations, in order to prevent evolution to the neurological stage. The peripheral neuropathy slowly leads to a progressive sensory loss that makes patients susceptible to inadvertent injuries, leading to foot ulcers, physical deformities, and grade-2 disabilities (G2D) [1,43,46]. Considering that refugees and immigrants are usually young, the prevention of the late complications by early diagnosis and treatment should be a priority.

Clinicians dealing with immigrants from endemic countries should at least be trained to the systematic identification of suspect skin lesions and to the palpation of peripheral nerves (more commonly ulnar and peroneal nerves) for enlargement or pain. Molecular diagnosis should be available in all referral centers, and research is also needed to identify useful markers of infections as well as of cure.

In conclusion, the diagnosis and management of leprosy in refugees and migrants from endemic countries is a challenge. We hope that this neglected disease will gain the necessary consideration among the health problems of refugees and migrants in Europe.

## Figures and Tables

**Table 1:** Demographic, epidemiologic, and clinical characteristics of 55 migrants diagnosed with leprosy reported from 2009 to 2018.

Characteristics	Total *n* = 55 (%)	Africa *n* = 23 (%)	Asia *n* = 22 (%)	Latin America *n* = 10 (%)
Male	37 (67.3)	18 (78.3)	17 (77.3)	2 (20)
Median age (IQR), Years	33 (28–41)	29 (23–37)	38 (28–43)	34 (28–41)
Median duration of stay in Europe (IQR), Months	36 (12–92)	21 (10–82)	55 (30–103)	35 (7–92)
Previous Diagnosis of Leprosy	19 (34.6)	10 (43.5)	2 (9.1)	7 (70)
Median Time from Start of Symptoms and Diagnosis (IQR), Months	12 (3–31)	26 (3–57)	5 (3–24)	11 (3–25)
Signs and Symptoms				
Skin	50 (90.9)	20 (87)	21 (95.5)	9 (90)
Neuropathy	39 (70.9)	19 (82.6)	14 (63.6)	6 (60)
Edema	4 (7.3)	2 (8.7)	2 (9.1)	0
Eye Involvement	3 (5.5)	1 (4.3)	2 (9.1)	0
Fever	3 (5.5)	2 (8.7)	0	0
Arthritis	2 (3.6)	0	1 (4.5)	1 (10)
Lymphadenopathy	2 (3.6)	1 (4.3)	1 (4.5)	0
Diagnosis				
Nasal Swab AFB Positive (*n* = 54) *	20 (37)	8/23 (34.8)	11/21 (52.4)	1/10 (10)
SSS AFB Positive (*n* = 54) *	39 (70.9)	13/22 (59.1)	18/22 (81.8)	8/10 (80)
Granulomatous Inflammation in the Biopsy (*n* = 43) ^	21 (48.8)	6/17 (35.3)	13/19 (68.4)	2/7 (28.6)
AFB positive in the biopsy (*n* = 43) ^	13 (30.2)	7/17 (41.2)	5/19 (26.3)	1/7 (14.3)
PCR for *M. leprae or M. lepromatosis* DNA positive (*n* = 24) §	17 (85)	7/9 (77.8)	8/9 (88.9)	2/6 (33.3)
WHO classification				
PB	19 (34.5)	10 (43.5)	5 (22.7)	4 (40)
MB	36 (65.5)	13 (56.5)	17 (77.3)	6 (60)
Ridley–Jopling classification				
TT	3 (5.4)	2 (8.7)	1 (4.5)	0
BT	27 (49.1)	12 (52.2)	9 (40.9)	6 (60)
BL	5 (9.1)	0	4 (18.2)	1 (10)
LL	17 (31)	8 (34.8)	6 (27.3)	3 (30)
BL/LL	3 (5.4)	1 (4.3)	2 (9.1)	0
Therapy				
MDT	52 (94.6)	20 (87)	22 (100)	10 (100)
ROM	3 (5.4)	3 (13)	0	0
Outcome				
Completed treatment	40 (72.7)	16 (69.6)	16 (72.7)	9 (90)
Ongoing	7 (12.7)	5 (21.7)	2 (9.1)	0
Lost	8 (14.6)	2 (8.7)	4 (18.2)	1 (10)

* data missing; ^ only patients who underwent a skin biopsy; § not done on all study population. **Abbreviations**: IQR, Interquartile range; PB, paucibacillary; MB, multibacillary; TT, tuberculoid leprosy; BT, borderline tuberculoid leprosy; BB, mid-borderline leprosy; BL, borderline lepromatous leprosy; LL, lepromatous leprosy; MDT, multidrug treatment; ROM.

**Table 2:** Epidemiologic characteristics of 243 migrants diagnosed with leprosy described in five national reports from 2009 to 2018.

Characteristic	Portugal (Medeiros S, 2009)	Greece (Kyriakis KP, 2010)	Italy (Massone C, 2010)	Denmark (Aftab H, 2016)	Spain (Ramos JM, 2016)
Years Analyzed	1968–2003	1988–2007	2002–2008	1980–2010	2003–2013
Number of Migrants/Total Patients (%)	36/102 (35.3)	6/33 (18.2)	58/64 (90.6)	15/15 (100)	128/168 (76.2)
Continent of Origin, *n* (%)					
Africa	20 (55.6)	2 (33.3)	16 (27.6)	2 (13.3)	27 (21.1)
South/Central America	11 (30.6)	1 (16.7)	19 (32.7)	0	92 (71.9)
Asia	5 (13.8)	3 (50)	23 (39.7)	13 (86.7)	7 (5.5)
Not Available	0	0	0	0	2 (1.5)

**Table 3:** Demographic, epidemiologic, and clinical characteristics of 37 migrants diagnosed with leprosy described in 14 case reports and three case series from 2009 to 2018.

Reference	Country (City)	Time	Gender Age (Years)	Country of Origin	Duration of Stay in Europe (Months)	Clinical Manifestations	Methods of Diagnosis	WHO Class	R-J Class
[24]	France (Saint-Brieuc)	2009	M, 11	Haiti	36	SNs (face), SPs (legs)	SB (AFB)	MB	LL
[7,25]	Italy (Genoa)	2006	M, 43	Brazil	NA	anesthetic and asymmetric SPs, erythematous SNs (abdomen, things and legs)	NB (AFB) SSS (AFB)	MB	BT
[26]	UK (Swindon)	2009	F, 23	Brazil	48	vascultic diffuse rash, fever, LA, SNs (face, arms and legs), polyarthritis with symmetrical synovitis (elbows, wrists, ankles, MCP joints)	SB (GI, AFB)	MB	LL, ENL
[27]	Italy (Palermo)	2009	M, 15	Senegal	6	anesthetic SPs, sensory loss and motor weakness (hand, forearm), claw deformity (hand), PN (ulnar, median nerves), paraesthesia (legs)	clinical	PB	TT
[7]	Italy (Genoa)	2010	M, 28	Nigeria	NA NA	SNs (face)	SB (AFB)	MB	LL
			M, 22	Colombia		SNs (extremities)	SB (AFB)	MB	NA
			M, 14	Brazil		symmetrical SPs (entire body)	SB (AFB)	MB	NA
[28]	Italy (Milan)	2010	M, 14	Brazil	96	erythematous SNs (face, extremities), SPs (arms), painful edema with hypoesthesia (wrists, hands), fever, LA, weight loss	SB (AFB, PCR)	MB	LL
[36]	Italy (Verona)	2006	M, 20	India	36	erythematous SNs and SPs (face, extremities), polyarthritis (wrists, ankles), fever, episcleritis	SB (MI, AFB)	MB	LL
[37]	Italy (Sassari)	2011	M, 26	Nigeria	12	SPs (trunk, extremities), symmetric edema (extremities), fever, headache, LA, PN (great auricular, ulnar nervs),	SB (GI)	MB	BB/BL
[38]	Spain (Malaga)	2004–2009	F, 28	Brazil	8	SP (harm)	SB (MI)	PB	I
			M, 33	Mali	8	SPs (extremities)	SB (MI)	PB	I
			F, 32	Nigeria	48	SP (trunk)	SB (MI)	PB	I
			F, 31	Paraguay	36	SPs (extremities)	SB (GI)	PB	TT
			F, 26	Brazil	12	SP (leg)	SB (GI)	PB	TT
			M, 40	Colombia	12	SPs (extremities, trunk)	SB (GI)	PB	BT
[29]	Spain (Alicante)	2011	M, 41	Colombia	108	painful edema (extremities), synovitis (wrists, MCP joints), tenosynovitis, SNs (legs), LA, mild skin rash on trunk	SB and LB (GI, AFB)	MB	LL, ENL
[30]	Spain (Valladolid)	2012	M, 19	Mauritania	48	SPs, sensory loss and motor weakness (extremities), claw deformity (hand)	SB (AFB)	MB	I
[31]	Italy (Ferrara)	2015	M, 22	Ghana	24	infiltration facial skin, pain, sensory loss and motor weakness (hand), ulnar palsy, erythematous SNs (face), arthralgia, arthritis (hands, ankles, knees), hair loss and madarosis	SB (AFB, GI)	MB	BT
[32]	UK (London)	2016	M, 60	Nigeria	36	facial weakness and numbness (extremities, face), thickened peripheral nerves, ulnar palsy, difficulty walking	NB (MI)	MB	PNL BT
[39]	Spain (Madrid)	1989	F, 40	Eq. Guinea	2 w	SP (face)	SB (GI)	PB	TT
		1990	M, 30	Philippines	48	SPs (face, extremities)	SSS (AFB)	MB	LL
		1994	M, 28	Eq. Guinea	96	erythematous SNs, PN	SSS (AFB)	MB	LL
		1996	M, 48	Colombia	12	hypoesthetic SPs	SSS/SB (AFB, MI)	MB	BB
		2002	F, 65	Colombia	12	infiltration facial skin, symmetrical PN	SSS/NB (AFB)	MB	LL
		2005	M, 59	DR	96	erythematous SNs	SSS (AFB)	MB	BB-BL
		2004	F, 62	DR	3	claw deformity (hands), PN legs, plantar ulcer	clinical	MB	LL
		2006	M, 24	Mali	12	SPs, asymmetric PN (left ulnar)	SB (GI)	MB	BB
		2006	F, 28	Brazil	24	infiltration facial skin, hypoesthesia legs	SSS (AFB)	MB	BL
		2007	M, 22	Brazil	12	SPs, thickening ear lobes/cheeks, fever, PN	SSS (AFB)	MB	LL
		2008	F, 32	Paraguay	84	asymmetric hypoesthetic SPs	SSS (AFB)	PB	BT
		2011	F, 27	Paraguay	12	painful SNs, fever, PN	SSS/SB (AFB, PCR)	MB	LL
		2013	M, 40	Venezuela	96	SPs, madarosis, PN	SSS (AFB)	MB	BL-LL
		2015	F, 38	Paraguay	60	SPs, PN (right ulnar)	SSS/SB (AFB, PCR)	MB	LL
[33]	UK (Birmingham)	2017	F, 15	Afghanistan	60	left ulnar palsy	clinical	MB	PNL
[34]	Germany (Homburg/Saar)	2016	M, 28	Afghanistan	NA	erythematous SNs and SPs (face, extremities)	SB (AFB, PCR, GI)	MB	NA
[35]	Italy (Rimini)	2017	M, 29	Nigeria	36	SNs (face, hands)	SB (AFB, PCR, GI)	MB	LL

**Abbreviations**: M, male; F, female; DR, Dominican Republic; SN, skin papule, plaque or nodule; SP, skin patch; PN, polyneuropathy; LA, lymphadenopathy; MCP, metacarpophalangeal; SB, skin-biopsy; SSS, slit-skin smear; NB, nerve-biopsy; LB, lymph node-biopsy; AFB, acid-fast bacilli; GI, granulomatous inflammation; MI, monocuclear infiltrate; PB, paucibacillary; MB, multibacillary; WHO class, WHO classification; R-J class, Ridley-Jopling classification; TT, tuberculoid leprosy; BT, borderline tuberculoid leprosy; BB, mid-borderline leprosy; BL, borderline lepromatous leprosy; LL, lepromatous leprosy; PNL, Pure Neuronal Leprosy; ENL, Erythema nodosum leprosum. NA, not available.

## Supplementary Materials

The following are available online at https://www.mdpi.com/2076-2607/8/8/1113/s1, Video S1: Deficit of abduction of the fifth finger.

## Appendix A

### Search Strategies (Electronic Searches)

A literature search was carried out to identify all possible studies that could help to answer the research question. The following databases were searched for relevant studies:

- Cochrane Central Register of Controlled Trials (CENTRAL 2018, Issue 7);
- MEDLINE (PubMed) (1966 to 14 August 2018);
- EMBASE (Embase.com) (1974 to 14 August 2018);
- Latin American and Caribbean Health Science Information Database (LILACS) (Bireme) (1982 to 14 August 2018);
- ClinicalTrials.gov (www.clinicaltrials.gov);
- World Health Organization (WHO) International Clinical Trials Registry Platform (apps.who.int/trialsearch); and
- Orphanet (www.orpha.net)
